# Delayed Erosion of Peritoneal Dialysis Catheter Tip into the Small Bowel

**DOI:** 10.7759/cureus.10806

**Published:** 2020-10-05

**Authors:** Abigail W Cheng, Mahmuod Abdeljaber, Saad Shebrain

**Affiliations:** 1 Surgery, Western Michigan University Homer Stryker MD School of Medicine, Kalamazoo, USA

**Keywords:** peritoneal dialysis, catheter tip erosion

## Abstract

Catheter erosion into the small bowel is an uncommonly reported complication of continuous ambulatory peritoneal dialysis (CAPD). This can result in peritonitis, sepsis, fistula formation, and mortality. We report a 29-year-old female with end-stage renal disease presenting with nausea, profuse diarrhea immediately after dialysate instillation, change in color and odor of the dialysis output, and pain at the site of her peritoneal dialysis (PD) catheter. After a thorough evaluation, catheter tip erosion into the patient’s small bowel lumen was diagnosed. This was confirmed when a blue bowel movement followed instillation of a saline solution with methylene blue into the dialysis catheter. A laparoscopic-assisted procedure was performed with removal of the dialysis catheter, resection of the diseased small bowel, and insertion of a right internal jugular tunneled dialysis catheter. Small bowel biopsy was benign. Post-operatively, the patient had no complications, was started on hemodialysis, and was discharged home.

## Introduction

Continuous ambulatory peritoneal dialysis (CAPD) is an attractive alternative to in-center hemodialysis as it grants patients relative independence. As with all operations, however, it does carry its own set of possible complications. The most serious complication is peritonitis, which is also the most common, with an average occurrence of once every 17.6 months per patient [1]. Other common complications include dialysate leaks, ultrafiltration failure, catheter failure, and hernias [2,3]. Erosion of the catheter tip into the bowel is a rarely reported complication of CAPD. More commonly, the erosion is into the large bowel rather than the small bowel [4]. Previous studies have not yielded a uniform trend with respect to risk factors predisposing patients to bowel erosion, nor have they demonstrated uniform presentation [4-9]. Quickly detecting and correcting bowel erosion in the setting of CAPD is imperative as this complication can result in peritonitis, sepsis, fistula formation, and death. We present a case of a 29-year-old woman who presented with peritoneal dialysis (PD) catheter erosion into the lumen of her ileum five months after the insertion of the catheter.

## Case presentation

Our patient is a 29-year-old Caucasian female with a history of hypertension, recurrent urinary tract infections (UTI), and end-stage renal disease. Seven years prior to presentation, she underwent right nephrectomy for an atrophic kidney secondary to recurrent nephrolithiasis. Five months prior to presentation, she had a PD catheter placed. Past surgical history also included three Cesarean sections and a laparoscopic cholecystectomy. She presented to the emergency department after four days of profuse diarrhea following dialysate instillation, as well as a decrease in PD output and change in output from clear to cloudy, brown, and foul-smelling. She had a persistent history of non-compliance and failure to follow up. She performed one to two manual PD sessions per day, typically putting in 2L and getting back 2L of clear, at times cloudy, fluid. She reported nausea and left lower quadrant abdominal pain at the site of her PD catheter, but denied any vomiting, hematochezia, fever, chills, chest pain, or shortness of breath. On physical exam, the patient was normothermic with stable vital signs, except for mild tachycardia at 104 beats per minute. Her abdominal exam showed a soft, non-distended abdomen with tenderness to the left lower quadrant around the catheter, but no peritoneal signs were noted. Dark brown, thick, foul-smelling material was visualized in the lumen of the catheter.

On admission, laboratory studies were significant for elevated blood urea nitrogen (BUN) at 38 mg/dL (normal range (NR) 6-20 mg/dL), elevated creatinine at 3.67 mg/dL (NR 0.60-1.10 mg/dL), decreased estimated glomerular filtration rate (GFR) of 15 mL/min (NR >60 mL/min), and CO2 19 mmol/L (NR 23-32 mmol/L). Urinalysis showed urine protein 100 mg/dL (NR negative), small amounts of blood (NR negative) and red blood cells (RBCs) 19/high-power field (HPF) (NR 0-2), all at patient’s baseline, and evidence of UTI with 3+ bacteria (NR negative), positive leukocyte esterase, and >100/HPF white blood cells (WBCs) (NR 0-2). Clean catch culture was positive only for normal urogenital flora.

**Figure 1:**
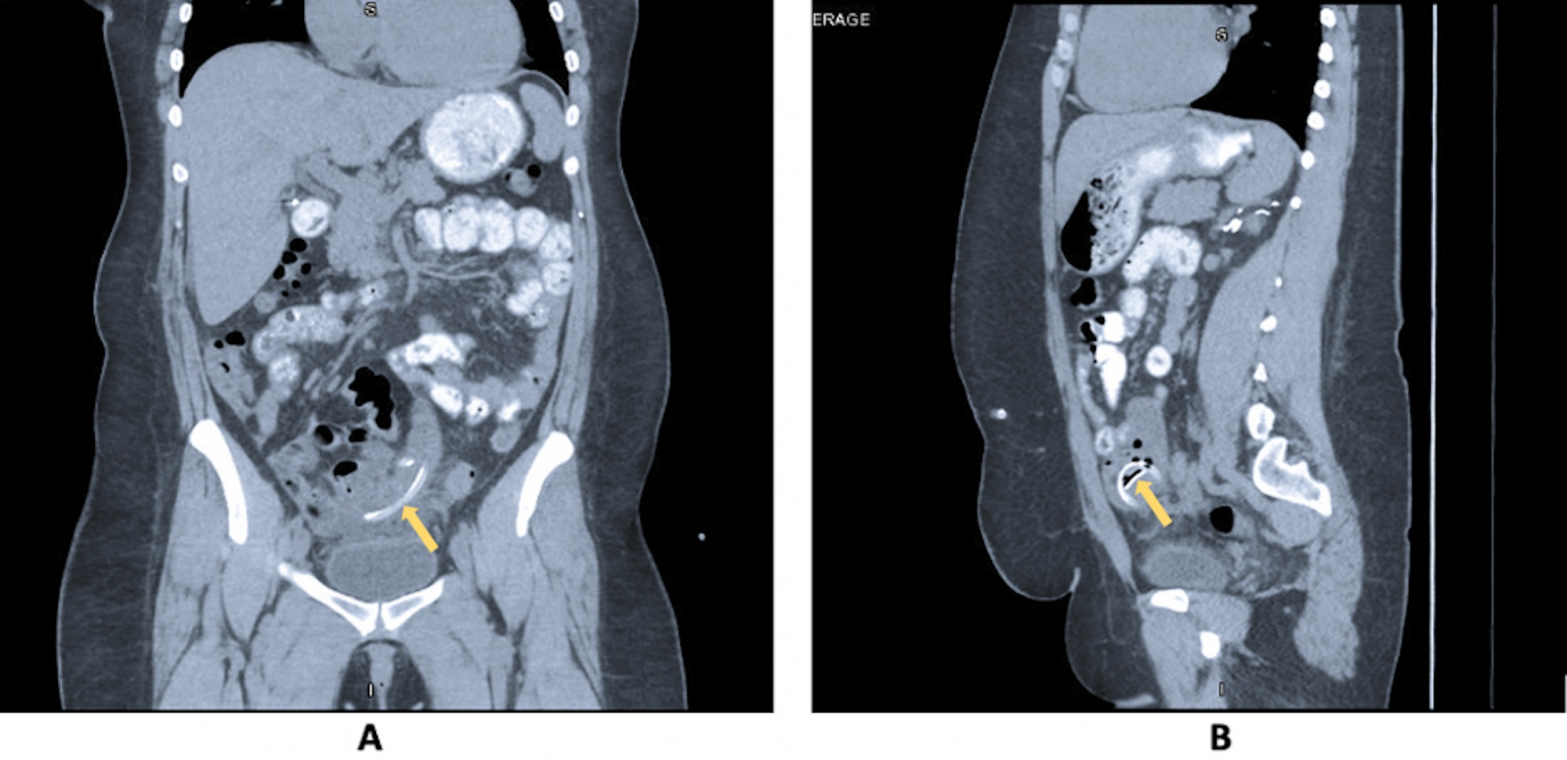
Abdomen/pelvis CT demonstrating peritoneal dialysis catheter appearing to be located within the lumen of the small bowel (yellow arrow, A, B).

**Figure 2:**
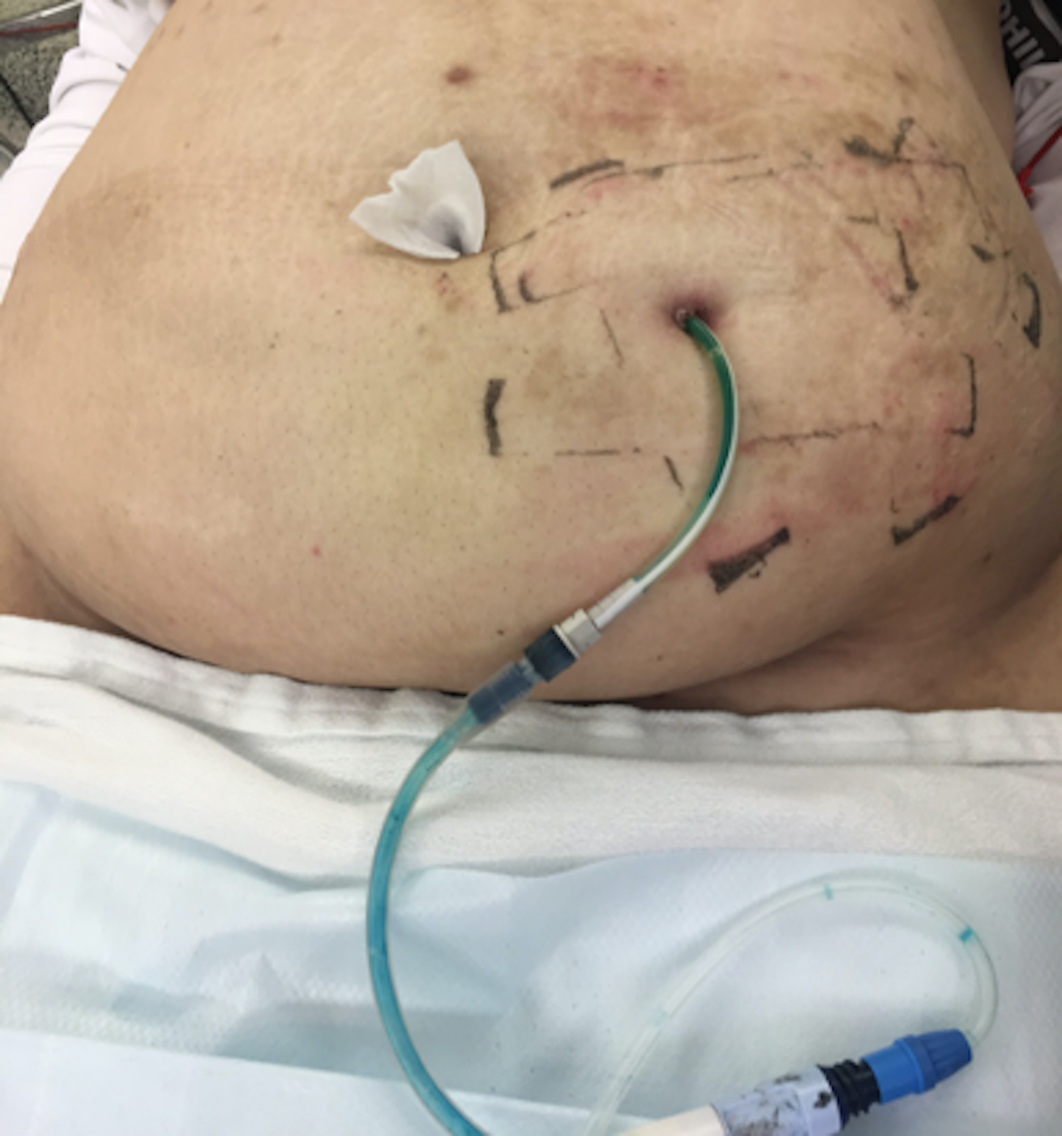
Mixture of 10 mL of methylene blue and 10 mL of saline into the peritoneal dialysis catheter.

Intraperitoneal vancomycin and ceftazidime were started on admission after PD catheter cultures were obtained. Computerized tomography (CT) scan of the abdomen and pelvis demonstrated surgically absent gallbladder and right kidney, and PD catheter overlying the left hemipelvis, appearing to be located within the lumen of the small bowel (Figures 1A-1B). Shortly after injecting a mixture of 10 mL of methylene blue and 10 mL of saline into her PD catheter followed by an 80 mL saline flush, the patient had a blue bowel movement, confirming the location of the PD catheter in the small bowel lumen (Figure 2). Her antibiotics were subsequently changed to intravenous (IV) vancomycin, cefepime, and fluconazole while awaiting culture results.

PD catheter culture was positive for Escherchia coli, Enterobacter cloacae, and Klebsiella pneumoniae, Streptococcus viridans group, Bacteriodes vulgatus, Bacteriodes distasonis, and mixed intestinal-type flora. The culture was also positive for normal skin flora, moderate WBCs, and yeast. Antibiotics were tailored to IV cefepime, fluconazole, and metronidazole. 

**Figure 3:**
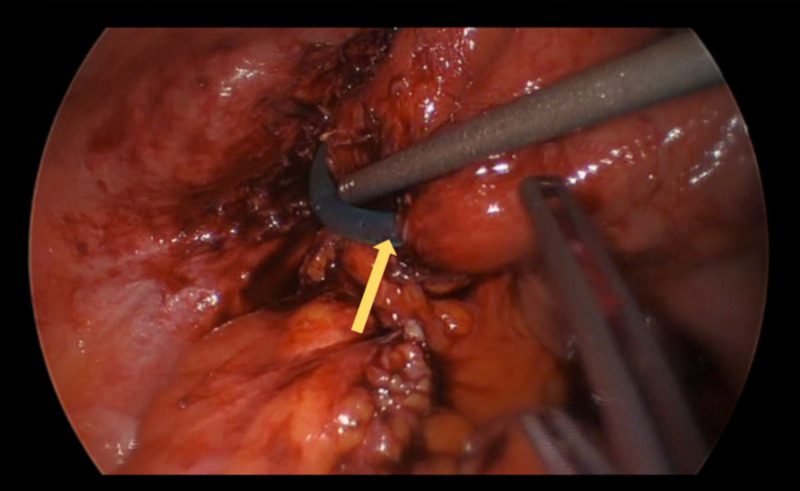
Site of catheter tip erosion into the small bowel (yellow arrow).

**Figure 4:**
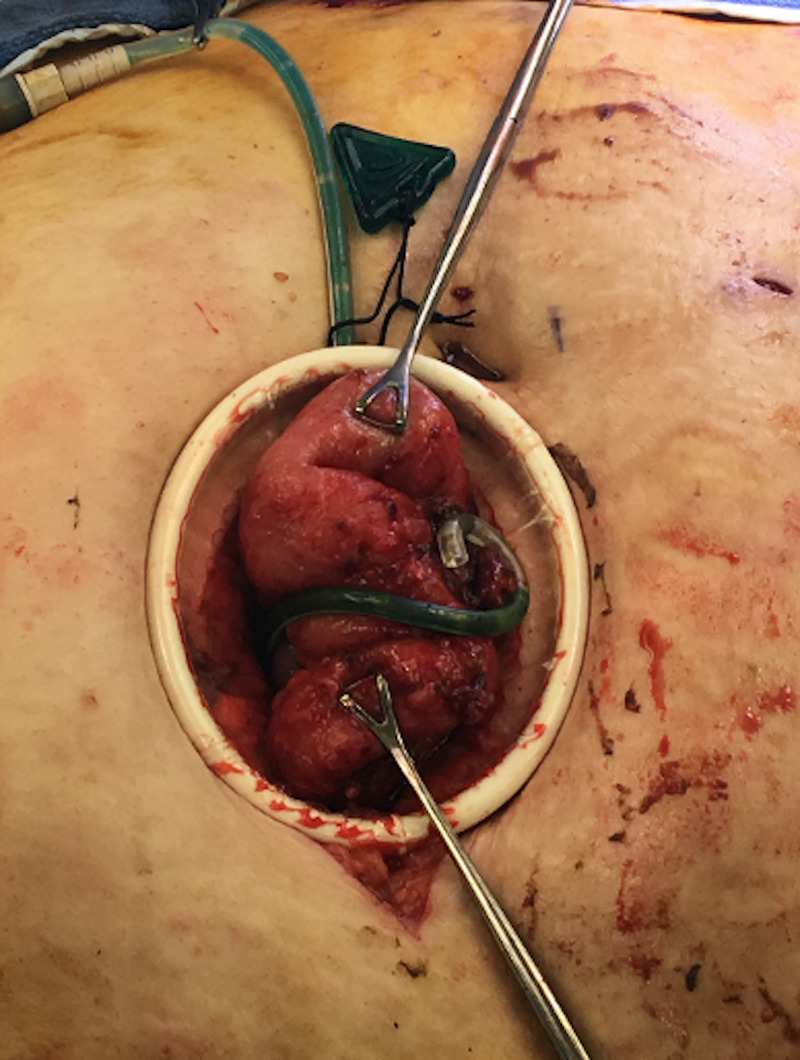
Exteriorization of the eroded catheter and affected small bowel prior to removal, resection and anastomosis.

She subsequently underwent a laparoscopic-assisted procedure with extensive adhesiolysis to identify the location of catheter tip erosion into the inflamed small bowel and subsequent removal of the dialysis catheter with resection of the diseased small bowel and primary anastomosis (Figures 3-4). A right internal jugular tunneled dialysis catheter was also inserted for hemodialysis. No suspicious findings were found on the small bowel biopsy.

## Discussion

Although bowel perforation during insertion of a CAPD catheter has a reported frequency of 0.7%-2.6% [10], delayed erosion of the catheter into the intestines is rarely reported. Accompanying symptoms often include diarrhea with dialysate inflow, peritonitis, and hematochezia [6]. However, a patient may present with no symptoms [6].

Standard diagnosis of PD catheter complications consists of catheterography, which uses water-soluble contrast injected into the PD catheter under fluoroscopy, or CT peritoneography [11]. These methods were not used in our case as our patient had residual intraluminal contrast from a CT abdomen/pelvis scan with oral contrast. Instead, we elected to inject methylene blue into the PD catheter while looking for any presence of blue dye in the stool. Although the use of methylene blue for diagnosis of PD catheter complications, such as bladder perforation, has been reported in the literature [12,13], this method has received criticism due to its potential for causing chemical peritonitis due to methylene blue’s potential irritant properties [14-17]. However, in cases like ours where CT peritoneography or catheterography would produce ambiguous results and suspicion for catheter tip erosion is high, we believe the benefits of methylene blue outweigh the risks and would be a suitable method for diagnosis.

Due to the heterogeneity of catheter erosions, diagnostic laparoscopy is a standard operative method [11]. This approach gave us visualization of a complex, looped, eroded catheter, which was valuable additional information not gleaned from the CT.

Risk factors predisposing patients to delayed intestinal erosion have not been well-described, but are hypothesized to include older age [5], large intestinal diverticulosis [18], and colonic amyloidosis [8]. Brady et al. hypothesized that continuous unlubricated contact of the catheter with the intestinal wall increases this risk [19]. Our patient is unique due to her younger age, location of the bowel erosion, and absence of any of the known reported risk factors.

## Conclusions

Catheter tip erosion into the small bowel is a rare complication in patients receiving PD. This can lead to peritonitis, sepsis, fistula formation, and death. The diagnosis should be considered in patients presenting with watery diarrhea following dialysate instillation, as well as changes in color and odor of the dialysis output. Initial workup with a complete blood count and catheter cultures are important in characterizing the severity of illness and to direct antibiotics. CT scan of the abdomen/pelvis is an important part of initial work up to obtain information regarding the location and structure into which the catheter has eroded. Subsequently, CT peritoneography and catheterography are standard protocol modalities for diagnosis and confirmation. In the event that these modalities are unavailable or will produce ambiguous results, such as if the patient has residual contrast from previous imaging, a mixture of methylene blue and saline can be injected into the dialysis catheter as an alternative. Finally, a diagnostic laparoscopy is performed to visualize the extent of erosion and affected tissue, and direct further management options.
